# Retinorecipient areas in the common marmoset (*Callithrix jacchus*): An image-forming and non-image forming circuitry

**DOI:** 10.3389/fncir.2023.1088686

**Published:** 2023-02-02

**Authors:** Nelyane Nayara M. Santana, Eryck H. A. Silva, Sâmarah F. dos Santos, Miriam S. M. O. Costa, Expedito S. Nascimento Junior, Rovena Clara J. G. Engelberth, Jeferson S. Cavalcante

**Affiliations:** ^1^Laboratory of Neurochemical Studies, Department of Physiology and Behavior, Bioscience Center, Federal University of Rio Grande do Norte, Natal, Brazil; ^2^Laboratory of Neuroanatomy, Department of Morphology, Bioscience Center, Federal University of Rio Grande do Norte, Natal, Brazil

**Keywords:** retinal projection, marmoset (*Callithrix jacchus*), image forming system, non-image forming system, retinorecipient areas

## Abstract

The mammalian retina captures a multitude of diverse features from the external environment and conveys them via the optic nerve to a myriad of retinorecipient nuclei. Understanding how retinal signals act in distinct brain functions is one of the most central and established goals of neuroscience. Using the common marmoset (*Callithrix jacchus*), a monkey from Northeastern Brazil, as an animal model for parsing how retinal innervation works in the brain, started decades ago due to their marmoset’s small bodies, rapid reproduction rate, and brain features. In the course of that research, a large amount of new and sophisticated neuroanatomical techniques was developed and employed to explain retinal connectivity. As a consequence, image and non-image-forming regions, functions, and pathways, as well as retinal cell types were described. Image-forming circuits give rise directly to vision, while the non-image-forming territories support circadian physiological processes, although part of their functional significance is uncertain. Here, we reviewed the current state of knowledge concerning retinal circuitry in marmosets from neuroanatomical investigations. We have also highlighted the aspects of marmoset retinal circuitry that remain obscure, in addition, to identify what further research is needed to better understand the connections and functions of retinorecipient structures.

## 1. Introduction

Afferents from the retina to the brain have been an important focus in connectional research for decades (Lane et al., 1971; Martin, 1986; Kaas and Huerta, 1988; Matteau et al., 2003). Although the geniculostriate circuitry has been the primary center of the research on retinal projection, it is long established that other retinorecipient nuclei and pathways exist, and over recent years a concerted effort to comprehend their functional significance has emerged (Kwan et al., 2018).

The body of work describing retinal innervation has relied on several animal models (Cassone et al., 1988; Murakami et al., 1989; Smale et al., 1991; Nemec et al., 2004; Scalia et al., 2014) to reveal the retinal terminal distribution (Guillery, 1970; Tigges and Tigges, 1981; Nascimento et al., 2010) and types of retinal fibers (Ling et al., 1997; de Góis Morais et al., 2014; Santana N. N. M. et al., 2018; Santana M. A. D. et al., 2018). Typical animal models for this research included non-human primates (Moore, 1973; Kaas et al., 1974; Tigges et al., 1977; Pinato et al., 2009), one of which is the common marmoset (*Callithrix jacchus*), which is a small Neotropical monkey, endemic to Northeastern of Brazil. Furthermore, progress in the knowledge of retinal circuitry has also been achieved by the refinement of the approaches for tracing (Salleeba et al., 2019). Pioneer studies used ablation combined with anterograde degeneration techniques (Campbell, 1969; Hendrickson et al., 1970; Scalia and Arango, 1979), while recent research has employed viral tracers (Martersteck et al., 2017; D’Souza et al., 2021). Even though these useful and sophisticated elements allow a connectional map of retinal inputs, the functional role of part of retinal projection remains unknown. Classically, image-forming (IF) and non-image forming (NIF) systems have been proposed to categorize a numerous of retinorecipient areas and retinal pathways (Cavalcante et al., 2005; Daneault et al., 2016). Here we described the retinorecipient areas with IF and NIF properties from neuroanatomical tracing techniques in common marmosets. This review is motivated by the recent emergence of this primate as a scientific model for studies of neural connections (Majka et al., 2016; Bakola et al., 2021), including research on retinal innervation. Marmosets are an already established animal model for brain research due to their unique neuroanatomy (Solomon and Rosa, 2014; Mitchell and Leopold, 2015; Fukushima et al., 2019; Morais et al., 2019; Ríos-Flórez et al., 2021), high reproductive efficiency, and small bodies (Okano et al., 2012; Ross, 2019). The renewed focus has been because, at least in part, of the successful generation of transgenic marmosets via lentiviral-mediated gene transfer (Sasaki et al., 2009) and by the development of gene-knockout marmosets via genome editing (Sato et al., 2016). Furthermore, to analyze and manipulate populations and networks in the marmoset brain, genetic approaches (MacDougall et al., 2016; Takaji et al., 2016; Mimura et al., 2021) and pipelines for the processing of neural anterograde tracer images (Abe et al., 2017; Lin et al., 2019; Skibbe et al., 2019) have become available. These genetic and anatomical techniques might will become new tools for neuroanatomical and functional investigations of retinal connections.

In the following sections, we will discuss the connectional criteria by which retinorecipient regions are categorized into distinct systems, as well as functional information that is still absent for the retinal innervation in multiple non-image-forming areas. Furthermore, to make this review more fluid, we will include references from other animal models to describe common morphological and functional characteristics of subcortical nuclei. In general, the comparative analysis can be found within those primary references. Several features of the subcortical nuclei of the marmoset are similar to those of other mammalian species, including rodents, macaques, and humans, and we will not emphasize them repeatedly.

## 2. Overall organization of the marmoset brain

Remarkably, the marmoset brain has several unique features that differentiated it from other primate species (Miller et al., 2016; Eliades and Miller, 2017; Hagan et al., 2017; Atapour et al., 2018), most notably the existence of an area 8C in premotor (Burman et al., 2015) and the lack of cortical area 44 (Roberts et al., 2007; Paxinos et al., 2012). At the same time, the marmoset brain shares common characteristics with other species within the primate order (Chaplin et al., 2013; Ghahremani et al., 2017), such as the dorsolateral prefrontal cortex, inferior temporal cortex, and dorsal pulvinar (Preuss, 2007). Another striking feature of this brain, similar to other primates, is the existence of the complex interconnected circuitry of subcortical areas that receive and process, both simultaneously and in parallel (Callaway, 2005; Nassi and Callaway, 2009). Obviously, the retina is the first step in this network, in which photic inputs are captured, transduced, and decomposed into multiple parallel pathways (Euler et al., 2014; Masland, 2017). These type of retinal signals are transmitted to the brain by diverse retinal ganglion cells (RGCs) (Martersteck et al., 2017). Each type of RGC is sensitive to distinct features of the external environment and conveys them via the optic nerve to retinorecipient areas (Masland, 2012), with IF and NIF functions (Sondereker et al., 2020). Bellow, we will briefly characterize IF and NIF circuits and describe the primary basis for the segregation between them.

## 3. Image forming and non-image forming circuits

Classically, the organization of retinal circuits has been divided into two functional branches, IF and NIF pathways (Seabrook et al., 2017; Sondereker et al., 2020). In this review, we have included the retinorecipient nuclei, that support vision indirectly in the IF circuitry. Our selection is based on the visuomotor features of these subcortical structures due to their involvement in the pupillary light reflex and involuntary eye movements to stabilize the image (see section 4.2).

The IF circuits give rise to vision directly. The high spatial and temporal resolution of IF pathways allows them to locate and perceive the shapes of objects, and their specific features, such as color, contrast, direction, and orientation (Sanes and Masland, 2015; Baden et al., 2016; Seabrook et al., 2017). The NIF circuits relay global luminance levels of the external environment to support photic-based modulation of core rhythmic physiological processes (Fu et al., 2005; Schmidt and Kofuji, 2009; Do and Yau, 2010; Daneault et al., 2016; Lazzerini Ospri et al., 2017; Seabrook et al., 2017), such as endogenous photoentrainment (Erkert, 1989; Wechselberger and Erkert, 1994; Glass et al., 2001; Silva et al., 2005), hormonal release (Sousa and Ziegler, 1998; Bertani et al., 2010), body temperature (Hetherington, 1978; Hoffmann et al., 2012), and sleep/awake cycle (Hoffmann et al., 2012), in addition to modulating the behavioral repertory for mating opportunities, foraging and predation (Vaze and Sharma, 2013).

Although several studies have been demonstrated interconnections across both IF and NIF circuits (Dacey et al., 2005; Estevez et al., 2012; Hannibal et al., 2014; Sonoda and Schmidt, 2016; Sondereker et al., 2020), the central element for the segregation between them is the partitioning of axonal projections from distinct classes of RGCs to different subcortical nuclei (Seabrook et al., 2017). It is known that RGCs project to (at least) 21 subcortical retinorecipient targets in the marmoset brain (Kaas et al., 1978; Costa et al., 1999; Cavalcante et al., 2005; Engelberth et al., 2008; Lima et al., 2012; de Sousa T. B. et al., 2013; Kwan et al., 2018), each of which exhibits a distinct functional role. The development of the connectomes pipeline for processing anterograde labeling data for the marmoset brain has opened the avenues for a rich understanding of retinal connectional patterns (Lin et al., 2019). Some of the retinorecipient structures, such as dorsal lateral geniculate nucleus (DLG) transmit retinal signals directly to the visual cortex, whereas others, such as superior colliculus (SC) indirectly connect to the cortex via intermediate nuclei, such as pulvinar or via feedforward pathways to the DLG. In addition, many (but not all) NIF structures receive cortical input. The functional significance of those cortical projections is uncertain. As far as we know, none of the hypothalamic retinorecipient centers, such as the suprachiasmatic nucleus (SCN), establish synaptic connections to the cortex.

Here, we do not characterize the organization, function, and homology of RGCs in marmoset retina. Previous publications (Ghosh et al., 1996; Goodchild et al., 1996; Wilder et al., 1996; Gomes et al., 2005; Jusuf et al., 2006; Eriköz et al., 2008; Szmajda et al., 2008; Masri et al., 2017) already provided an excellent in-depth description of the marmoset RGCs population. Our objective is to provide an overview of IF and NIF retinorecipient targets in the marmoset brain in order to demonstrate that there is a substantial body of knowledge regarding the retinal innervation pattern. This part of its functional characteristics still needs examination.

## 4. Image forming system

**FIGURE 1:**
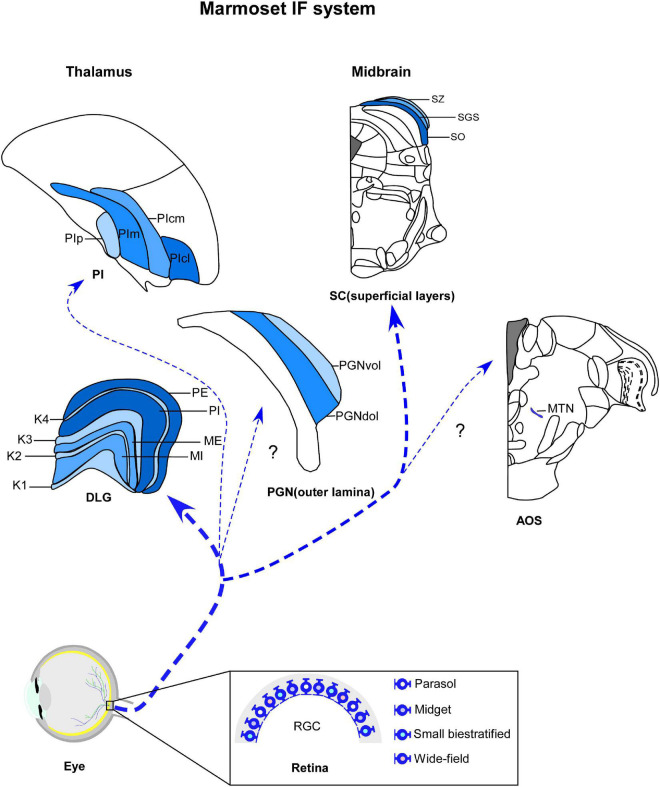
Schematic representation of marmoset image-foming system (blue). Distinct types of retinal ganglion cells (RGCs) bilaterally innervate visual thalamic and midbrain nuclei, such dorsal lateral geniculate nucleus (DLG), inferior pulvinar (PI), outer lamina of the pregeniculate nucleus (PGNol) and superficial layers of superior colliculus (SC). Parasol, midget and small bistratified cells send axonal projections to parvocellular, magnocellular and koniocellular layers of DLG, respectively. SC receives dominant projections from parasol cells. Additional wide-field cells, such as narrow thorny, broad thorny and recursive cell has been described as sending projections to K laminae, CS and PI. The RGC populations that innervates the PGNol, pretectal nuclei and accessory optic nuclei (AOS) still remain unclear. K1–K4, koniocellular layers 1–4; ME, external magnocellular layer; MI, internal magnocellular layer; PE, external parvocellular layer; PGNdol, pregeniculate nucleus dorsal outer lamina; PGNvol, Pregeniculate nucleus ventral outer lamina; PE, internal parvocellular layer; PIcl, central lateral nucleus of the inferior pulvinar; PIcm, central medial nucleus of the inferior pulvinar; PIm, medial nucleus of the inferior pulvinar; Pip, posterior nucleus of the inferior pulvinar; PM, medial pulvinar; PL, lateral pulvinar.

Anatomically, the IF circuitry is composed of a series of structures from the retina to the visual cortex, passing through several thalamic and midbrain nuclei (Daneault et al., 2016; Figure 1). In the first part of this review, we will focus on the subcortical nuclei of this system, which receive retinal afferents and exhibit predominantly, or exclusively IF functional properties, including visuomotor features.

### 4.1. Thalamus

The IF thalamus is a collection of subcortical nuclei that receives, processes, and transmits information about the visual scene to the cortex (Kerschensteiner and Guido, 2017), in addition to supporting a visuomotor behaviors (Livingston and Fedder, 2003). The three main visual thalamic nuclei are the DLG, pulvinar complex (Sherman and Guillery, 2002; Saalmann and Kastner, 2011) and pregeniculate nucleus (PGN) (Moore, 1989, 1993). These structures are segregated based on their connectional, neurochemical, and functional patterns (Sherman, 2016). The DLG and pulvinar are retinorecipient components of the dorsal thalamus and comprise the two main, functionally distinct visual (geniculostriate and extrastriate) pathways, by which retinal information reaches multiple visual cortices (Harting et al., 1973; Casagrande and Khaytin, 2009). In contrast, the dorsolateral lamina of PGN, considered the non-primate homologous to the ventral lateral geniculate nucleus (VLG) (Moore, 1989, 1993; Lima et al., 2012), is the target of retinal afferents in the ventral thalamus and may modulate the gaze control (Livingston and Mustari, 2000; Livingston and Fedder, 2003). Here, we will highlight the retinorecipient thalamic nuclei involved with IF circuits in marmosets (Figure 1).

#### 4.1.1. Dorsal lateral geniculate nucleus (DLG)

Traditionally, the DLG is a well-established retinorecipient thalamic station that relays image-forming visual input from the retina to primary visual cortex (V1) (Solomon and Rosa, 2014; Mitchell and Leopold, 2015), albeit subsequent studies have suggested a more complex functional role of this structure (see Dan et al., 1996; O’Connor et al., 2002; Belluccini et al., 2019; Dougherty et al., 2019; see Weyand, 2016, for a description of multifunctional nature of the DLG). Based on its relay function, which conveys peripheral (“driver”-type) information to the cortex along the retinocortical pathway, the DLG is considered a first-order thalamic nucleus (Sherman and Guillery, 1998; Bickford, 2016). In addition to retinal innervations that comprises only a minority of the synaptic input to the DLG (de Sousa A. A. et al., 2013), this nucleus receives projections from visual cortices, the thalamic reticular nucleus, and visuotopically organized subcortical structures (Hubel and Wiesel, 1961; Cleland et al., 1971; Usrey et al., 1999; Zeater et al., 2018). Therefore, the DLG represents the first stage of visual processing due to its modulatory influence on visual information before conveying it to the visual cortex (Kaas and Huerta, 1988; Litvina and Chen, 2017).

The DLG of marmosets has a laminar profile, similarly to other primates, with parvocellular (P), magnocellular (M), and koniocellular (K) neurons segregated in multiple and functionally distinct layers (Spatz, 1978; Fitzpatrick et al., 1983; de Sousa A. A. et al., 2013; Baldwin and Krubitzer, 2018; Figure 1). Each cell type differs dramatically in terms of their morphological, physiological, and connectional features, representing distinct parallel channels for visual processing (Mitchell and Leopold, 2015). The P pathways provide high-acuity vision and red-green color vision (Lennie and Movshon, 2005; Martin and Grünert, 2013), while the visual inputs conducted by the M channel provide for spatial and motion analysis (Lee et al., 2010; Percival et al., 2014). A third pathway involves cells located in the K layers, which form another chromatic channel that mediates blue/yellow opponency discrimination (Wässle, 2004; Szmajda et al., 2006, 2008; Roy et al., 2009). In the DLG of marmosets, through classical architectural procedures, such as Nissl and hematoxylin, it is possible to recognize two P layers (internal—PI and external—PE), two M layers (internal—MI and external—ME), and four K laminae (K1–K4) (Kaas et al., 1978; Spatz, 1978; Warner et al., 2010).

Connectional studies provided clear evidence of the retinorecipient nature of the marmoset DLG (Kaas et al., 1978; Lima et al., 2012; Kwan et al., 2018). In common with other mammalians, RGC axons project in an orderly anatomically manner to marmoset DLG (Kaas et al., 1978; Kaas and Huerta, 1988; White et al., 1998; Kwan et al., 2018). The patterning of retinogeniculate projections involves retinal topography, ocular map and lamination specificity of RGCs classes (Pfeiffenberger et al., 2005; Huberman et al., 2008).

*Retinotopy.* The RGCs send axonal projections to the DLG in an orderly fashion to preserve spatial information about the visual scene, forming retinotopic maps (Grubb and Thompson, 2003; Piscopo et al., 2013; Guido, 2018). Our comprehension of the retinotopic organization in the DLG of marmosets arises from an electrophysiological experiment performed by White et al. (1998). In this report, using extracellular recordings from the DLG neuronal responses, they showed that the contralateral hemifield is represented in this structure (White et al., 1998). Furthermore, these researchers demonstrated that, within each DLG layer, the dorsal visual field is represented laterally, and the ventral visual field is represented medially. The representation of the central foveal vision is located posterodorsally within the DLG, with the peripheral vision progressing anteroventrally (White et al., 1998). This visuotopic pattern is similar to those described for the DLG of other primates.

The mechanisms underlying the retinotopic order have been well studied, and much of our current knowledge may be attributed to the use of transgenic mouse models (Huberman et al., 2008; Kim et al., 2010; Cang and Feldheim, 2013). These reports reveal the cellular and molecular events and drive the refinement of the retinotopy of the DLG, including axon mapping, arbor pruning, neural activity, synapse elimination, and Eph/ephrin signaling (Hong and Chen, 2011; Ackman et al., 2012). However, the targeting mechanisms of retinogeniculate projections in marmosets remain unexplained.

*Eye-specific and binocular projections* Several electrophysiological (Warland et al., 2006; Zhang et al., 2012), molecular (Pfeiffenberger et al., 2005; Nakamoto et al., 2018), and anatomical (Godement et al., 1984; Muir-Robinson et al., 2002; Jaubert-Miazza et al., 2005) reports have strongly supported the canonical principle of eye-specific segregation of the RGCs axons in the DLG. According to this principle, retinal afferents are separated into distinct DLG laminae and occupy non-overlapping domains (Wallace et al., 2016). In carnivores and primates, each DLG layer receives visual monocular input from the ipsilateral or contralateral eye (Wiesel and Hubel, 1966; Casagrande and Norton, 1991). However, binocular responses or interactions were already has been reported in the DLG of mammalians (Erulkar and Fillenz, 1960; Xue et al., 1987). Binocular responses have been described in monkeys and cats in this structure (Sanderson et al., 1971; Rodieck and Dreher, 1979; Xue et al., 1987), albeit these interactions are naturally suppressive (Sanderson et al., 1971) and require conditions of specialized stimulus (Sanderson et al., 1969, 1971). In rodents, such as rats and mice, the DLG does not exhibit a discernible lamination, the retinal projections from both eyes are only partially segregated (Reese, 1988; Leamey et al., 2007) and many cells have binocular innervation (Grieve, 2005; Rompani et al., 2017). Similarly, it has been demonstrated that the all K layers of marmoset DLG receive a binocular input, although K1 and K3 layers showing columns of ocular segregation (Cheong et al., 2013; Zeater et al., 2015; Kwan et al., 2018).

In general, the organizational pattern of retinogeniculate projections in marmosets is similar to that seen in all primates studied as revealed by anterograde labeling techniques. The retinal inputs in marmoset DLG are topographically organized and delineated in a laminar pattern (White et al., 1998; Kwan et al., 2018). The P and M external laminae receive input from the contralateral nasal retina, whereas P and M internal layers are innervated by the ipsilateral temporal retina (Kaas et al., 1978; Kaas and Huerta, 1988; Kwan et al., 2018). In contrast to monocular excitatory responses of neurons in P and M layers, connectional and physiological studies demonstrated that the subset of K cells exhibits binocular responses (Cheong et al., 2013; Zeater et al., 2015; Kwan et al., 2018). Cheong et al. (2013) and Zeater et al. (2015), recorded single-cell activity in the DLG of anesthetized marmosets, and revealed that the subpopulation of K neurons showed vigorous excitatory response evoked through stimulation of either eye (Cheong et al., 2013 and Zeater et al., 2015). Although autoradiographic evidence has shown that K3 receives a retinal contralateral projection and K1 is innervated bilaterally by the retina (Spatz, 1978), anatomical studies, using bidirectional tracers, demonstrated that all K layers are a target of binocular input, with K1 and K3 laminae exhibiting alternating columns of ipsilateral and contralateral inputs (Kwan et al., 2018). It is speculated that connections from K layers with midbrain nuclei, that regulate spatial attention and orienting, such as CS and parabigeminal nucleus (Casagrande and Kaas, 1994; Hendry and Reid, 2000), potentially provide an indirect route to the V1 (Hendry and Yoshioka, 1994; Solomon et al., 2002; Casagrande et al., 2007) and visual association cortices (Sincich et al., 2004; Warner et al., 2010) used for higher-level form and motion analysis (Zeater et al., 2015). However, the functional and evolutionary role of binocular convergence in the K layers of the marmoset still needs to be extracted.

Numerous studies have shown that activity-mediated binocular competition (Lund et al., 1974; Chalupa and Williams, 1984; Guillery et al., 1985), remodeling of synaptic connections (Chen and Regehr, 2000; Guido, 2008; Hong and Chen, 2011), and retinal waves (Katz and Shatz, 1996; Cohen-Cory, 2002; Torborg and Feller, 2005; for review, see Thompson et al., 2017) play an instructional role in the formation of eye-specific retinogeniculate axons and retinotopic maps in several species of mammalians (Assali et al., 2014). In marmosets, these mechanisms of refinement of retinal circuits in the DLG remain unclear.

*RGC class-specific projections*. Morphological and connectional reports reveal diversity among RGCs, which comprise (at least) 17 distinct cell types in the retina of marmosets (Ghosh et al., 1996; Szmajda et al., 2005, 2008; Jusuf et al., 2006; Masri et al., 2017). These anatomical works demonstrate that subset of these RGCs types selectively project to the DLG, suggesting that parallel retinal signals enter it and remain segregated within this structure (Kaas and Huerta, 1988; Lee et al., 2010).

Consistent with previous findings in macaques, DLG layers of the marmoset receive input from specific classes of RGCs. Although melanopsinergic intrinsically photosensitive RGCs (ipRGCs), a heterogenous subpopulation that mediate NIF functions, send axonal projections to the DLG (Szmajda et al., 2008), the three best-understood retinogeniculate pathways originate from the parasol, midget, and small bistratified cells (Martin and Grünert, 2013). Most of the RGCs are of the midget type, and project to P layers of the DLG (Goodchild et al., 1996; Gomes et al., 2005; Jusuf et al., 2006). These cells are described as having sustained responses to photic stimuli (Ghosh and Grünert, 1999), selectivity to chromatic (red/green) signals (Martin and Grünert, 2013), and a small soma with a single primary dendrite that branches densely into small dendritic fields, as a main morphological feature (Szmajda et al., 2005; Masri et al., 2017). The second largest class of RGCs is that of parasol cells (Chan et al., 2001; Gomes et al., 2005; Eriköz et al., 2008), which are morphologically characterized by two-four dendrites emerging from a large soma, forming a large branched dendritic tree (Szmajda et al., 2005; Masri et al., 2017). They innervate the M laminae of the DLG (Szmajda et al., 2008), exhibit transient responses to photic input (Ghosh and Grünert, 1999), and contribute to motion perception and spatial vision at low image contrast (Szmajda et al., 2005). Small bistratified cells have synaptic connectivity with K layers of the DLG, particularly K3 (Szmajda et al., 2008), strong blue on/yellow off-color sensitivity (Martin et al., 1997), and relatively small dendritic field diameters (Szmajda et al., 2008; Masri et al., 2017; Paknahad et al., 2021).

Connectional works on the DLG of marmosets has demonstrated that other types of RGCs project to the K layers, albeit their physiological and functional characteristics are less well defined (Szmajda et al., 2008; Percival et al., 2013). Retrograde labeling techniques show that the K1 is a preferential target of narrow thorny cells (Percival et al., 2014). Based on the connectional pattern of K1 with the extrastriate regions, it is suggested that thorny-koniocellular circuitry takes part in residual visual capabilities (“blindsight”) following lesions of V1 in adult or early life (Rodman et al., 1989; Rosa and Tweedale, 2000; Percival et al., 2014). Furthermore, it has also been reported that broad thorny and recursive cells send sparse axons to the K3 lamina (Szmajda et al., 2008; Percival et al., 2011, 2013).

Despite the recent progress in the description of the marmoset retinogeniculate circuitry, the projection patterns of several classes of RGCs and their functional role are still unknown. The morphological diversity in the RGCs of marmosets (Szmajda et al., 2005, 2008; Jusuf et al., 2006; Moritoh et al., 2013; Masri et al., 2017) and their homology with RGCs of other species (Dacey, 2004; Berson et al., 2010; Sanes and Masland, 2015) have been scrutinized previously and, therefore, will not be addressed here. However, whether the connectional pattern of wide-field cells (non-midget, non-parasol, and non-small bistratified cells) in the retina of marmosets shows the same diversity in their retinorecipient nuclei as has recently been reported for the RGCs population in non-primates, especially for mice (Dhande and Huberman, 2014; Robles et al., 2014; Gauvain and Murphy, 2015; Ellis et al., 2016), will require further analysis.

#### 4.1.2. Inferior pulvinar (PI)

The pulvinar complex, referred to as the lateral posterior nucleus in non-primates, is a higher-order thalamic nucleus with multimodal properties, which harbors visually responsive neurons (Kaas and Lyon, 2007; Kwan et al., 2018). Functionally, the pulvinar has been implicated in modulating of visual attention (Chalupa et al., 1976; Bender, 1982; Petersen et al., 1985; Robinson et al., 1986); integration of sensory and cognitive signals (Bridge et al., 2016); shaping of the functional organization of the extrastriate cortex, particularly during early development (Bridge et al., 2016); and regulating cortico-cortical communication (Jones, 2001; Sherman and Guillery, 2002; Shipp, 2003; Saalmann and Kastner, 2011).

Based on the descriptive analysis of chemoarchitectural (Cusick et al., 1993; Stepniewska and Kaas, 1997; Baldwin et al., 2011, 2013; Balaram et al., 2013) and anatomical studies (Warner et al., 2010; Kwan et al., 2018), the pulvinar complex is traditionally subdivided into anterior (oral) medial, lateral, and inferior nuclei (Olszewski, 1952; Kaas and Huerta, 1988; Kaas and Lyon, 2007; Homman-Ludiye and Bourne, 2019; Froesel et al., 2021). The two former nuclei exhibit multisensory (Barbas and Mesulam, 1981; Baleydier and Morel, 1992; de la Mothe et al., 2006, 2012) and somatosensory functions (Mesulam et al., 1977; Acuña et al., 1983), whereas the latter ones, collectively known as visual pulvinar, are dedicated to visual processing and contain a retinotopic map of the contralateral visual hemifield, as well as strong connections to the visual cortex and from the SC (Kaas and Huerta, 1988; Baleydier and Morel, 1992; Stepniewska, 2003; Kaas and Lyon, 2007; Kaas and Baldwin, 2019; Moore et al., 2019). However, only portions of the inferior pulvinar (PI) are also recipients of retinal projections (Benevento and Standage, 1983; Nakagawa and Tanaka, 1984).

The PI has functionally distinct areas, with differences in neuropeptidergic and connectional patterns (Lin and Kaas, 1980; Cusick et al., 1993; Gutierrez et al., 1995; Stepniewska and Kaas, 1997; Cola et al., 1999; Gray et al., 1999; Adams et al., 2000). Despite some divergence in the terminology used to categorize the PI subdivisions (Mathers, 1971; Spatz, 1975; Gutierrez et al., 1995), we have kept the terms adopted by Stepniewska and Kaas (1997), such as medial nucleus (PIm), posterior nucleus (PIp), central medial nucleus (PIcm) and central lateral nucleus (PIcl) of the inferior pulvinar (Figure 1; Stepniewska and Kaas, 1997; Kaas and Lyon, 2007; Kaas and Baldwin, 2019). In general, the PIm is the major target of retinal afferents in the primate pulvinar (Kaas and Lyon, 2007; Baldwin and Bourne, 2017). In combination with PIp and PIcm, it sends axonal projections to dorsal stream visual areas for visually guided actions, whereas the PIcl is mainly devoted to the ventral stream of cortical processing for visual perception (Kaas and Lyon, 2007; Kaas and Baldwin, 2019).

As in all primates studied so far, a retinopulvinar projections in marmosets has been documented (Warner et al., 2010; Kwan et al., 2018). Anatomical reports identified contralateral retinal terminations that are sparse and primarily restricted to PIm (Warner et al., 2010; Kwan et al., 2018), with a few scattered retinal inputs supplying the PIcm and PIcl (Kwan et al., 2018). These studies also show very sparse ipsilateral retinal projections in PIm, in addition to sparser terminals along the boundaries PIp, PIcm, and PIcl (Warner et al., 2010; Kwan et al., 2018). Furthermore, one of those works also reveals, through co-injections of bidirectional tracers, the RGCs subtypes that are the source of these retinal projections to PIm (Kwan et al., 2018). Contrary to a previous report in macaques (Cowey et al., 1994), the subpopulation of RGCs that innervates the PIm of marmosets is that of wide-field cells, mainly broad thorny cells, along with recursive bistratified, narrow thorny and large bistratified cells (Kwan et al., 2018; Grünert et al., 2021). Further studies are needed to discover if other classes of RGCs innervate different regions in the PI of marmosets.

Over the last four decades, considerable progress has been made in understanding the retinotopic organization of the primate pulvinar (Campos-Ortega and Hayhow, 1972; Gattass et al., 1978; Bender, 1981; Petersen et al., 1985; Baldwin et al., 2011; Li et al., 2013). Connectional and electrophysiological reports show it contains two retinotopic maps of the contralateral visual hemifield in its lateral and inferior subdivisions. Their positions and visual field representations exhibit some species-specific singularities (Gattass et al., 1978; Bender, 1981; Li et al., 2013). In marmosets, the visuotopic order of the pulvinar has not yet been investigated in any detail.

#### 4.1.3. Outer lamina of the pregeniculate nucleus (PGNol)

The PGN is a retinorecipient structure of the ventral thalamus topographically dorsal and medial to the DLG (Livingston and Mustari, 2000; Livingston and Fedder, 2003). The prominent neurochemical content and anatomical connections of the PGN laminae with the retina (Babb, 1980; Moore, 1989, 1993; Costa et al., 1998; Pinato et al., 2009; Lima et al., 2012) and IF and NIF subcortical nuclei (Hendrickson et al., 1970; Mustari et al., 1994; Büttner-Ennever et al., 1996b; Chevassus-Au-Louis and Cooper, 1998; Kwan et al., 2018) suggest that it contributes significantly to visuomotor activities and circadian rhythmicity.

Traditionally, the PGN has been described as a laminar structure showed distinct regions with respect to retinal innervation patterns, functional role, and cytoarchitecture (Moore, 1989, 1993). These subsectors include (1) a large region located dorsomedially to the DLG, continuous with the zona incerta, which contains neuropeptide Y (NPY)-ergic neurons and dense retinal innervation, and (2) subdivision contain a scattered neuronal cluster located dorsal and lateral to the DLG (Moore, 1993; Costa et al., 1998; Pinato et al., 2009), that is contiguous with the reticular thalamic nucleus and sparsely receives retinal projections (Moore, 1989, 1993; Costa et al., 1998; Pinato et al., 2009). Despite the divergent nomenclatures of the PGN divisions (Hendrickson, 1973; Babb, 1980; Livingston and Mustari, 2000), its inner portion of PGN (PGNil) is considered equivalent to the intergeniculate leaflet (IGL) of non-primates, a modulating structure of the circadian timing system (CTS) (Moore, 1993; Costa et al., 1998; Pinato et al., 2009) that will be discussed in the next sections. The PGNol, by contrast, is likely the primate counterpart to the VLG (Moore, 1989, 1993; Costa et al., 1998; Lima et al., 2012).

Although more experimental approaches are needed to define the anatomical organization and functional role of the PGN of marmosets in the IF circuitry, its outer portion (PGNol) (Figure 1) has been proposed as a structure equivalent to the VLG (Lima et al., 2012), based on neuropeptidergic content, cell morphology, and connectional patterns with the retina (Costa et al., 1998; Lima et al., 2012) and pulvinar (Kwan et al., 2018). Here, we will follow this classification for a more complete characterization of the PGN of the marmoset.

As expected from reports on other species of primates, anterograde tract tracing has shown the bilateral retinal innervation in the PGN of marmosets (Costa et al., 1998; Lima et al., 2012). Cholera toxin B subunit (CTb)-labeled retinal fibers and terminals project sparsely to the PGNol, with contralateral predominance (Lima et al., 2012). In the ipsilateral side, the ventral portion of PGNol (PGNvol) exhibits a lower density of retinal terminal arbors compared to PGNil, whereas the dorsal part (PGNdol) is poorly innervated (Figure 1; Lima et al., 2012). These results suggest that PGNvol and PGNdol are equivalent to the external and internal portions of the VLG, respectively (Lima et al., 2012).

There has been limited investigation of the classes of RGCs that project to the PGN (Cowey et al., 2001; Hannibal et al., 2014). In macaques, neural tracer injections demonstrated that midget cells predominantly project to the PGN, although terminals from other RGCs subtypes were also identified (Cowey et al., 2001; Hannibal et al., 2014). In marmosets, no study has yet investigated the typology of RGCs that comprise the retina-PGN pathway.

Our knowledge about the visuomotor nature of the PGN comes mainly from ablation-behavioral evidence (Polyak, 1957) and electrode recordings of the PGN neuronal responses of macaques to visual stimuli (Büttner and Fuchs, 1973; Magnin and Fuchs, 1977; Livingston and Fedder, 2003). Although it was initially proposed that the PGN participates in the pupillary light reflex (Polyak, 1957), electrophysiological evidence revealed that it is involved in the modulation of gaze control; including saccadic movements, pursuit smooth eye movements, and visual motion or eye position (Büttner and Fuchs, 1973; Magnin and Fuchs, 1977; Livingston and Fedder, 2003); indicating its functional homology with the VLG. There are no equivalent reports for marmosets.

### 4.2. Midbrain

In different animal species, the IF midbrain (Figure 1) comprises several nuclear populations that mediate visuomotor reflexes (Gamlin, 2006; Giolli et al., 2006). Although functional and connectional similarities between some of these nuclei have been described (Simpson, 1984; Hoffmann et al., 1988; Simpson et al., 1988; Mustari et al., 1994), cytoarchitectonic evidence and other hodological connections have shown that there are distinctions in several mesencephalic nuclei (Gregory, 1985; Lui et al., 1995; Büttner-Ennever et al., 1996a,b), segregating them into different oculomotor subsystems. The most extensively studied midbrain nuclei are the SC, pretectal complex, and accessory optic system (AOS). The SC translates sensory inputs into motor outputs to guide innate behavior (Ito and Feldheim, 2018). The pretectal nuclei, such as the nucleus of the optic tract and the pretectal olivary nucleus, play a significant role in the optokinetic nystagmus (Hoffmann and Distler, 1989; Mustari and Fuchs, 1990) and pupillary light reflex (Pong and Fuchs, 2000; Szkudlarek et al., 2012). The accessory optic system has functional significance in the detection of retinal slip signals and relaying them to the oculomotor circuit for image stabilization (Fredericks et al., 1988; Masseck and Hoffmann, 2009; Lilley et al., 2018). As far as we know, systematic studies on the retinal projection to the pretectal complex in marmosets are needed. In addition, the typology of RGCs that comprise these retina-midbrain pathways has also not been completely elucidated. As we argued above, these limitations become particularly obvious when one considers the discussion of the retinal projection in the midbrain. Therefore, in this next section, we will explore the connectional pattern of the retina with the SC and AOS.

#### 4.2.1. Superior colliculus (SC)

The SC, also known as the optic tectum in non-mammalians, is a multimodal integrative hub for mediating sensorimotor transformations (Sparks and Mays, 1990; Stein and Meredith, 1993; Hall and Moschovakis, 2004; Chong et al., 2022). Although higher cognitive functions are attributed to the SC (Basso and May, 2017), its two main functional roles are convey retinal signals to other subcortical visual nuclei (Kaas and Huerta, 1988; May, 2006; Basso and May, 2017) and integration of multimodal stimuli into motor commands for orienting movements, and to redirect attention (Gandhi and Sparks, 2003; Gandhi and Katnani, 2011; Basso and May, 2017; Villalobos et al., 2018; Farrow et al., 2019).

Residing on the roof (tectum) of the midbrain, the SC has a laminar profile with seven layers (Kaas and Huerta, 1988; Gandhi and Katnani, 2011; Timurkaan et al., 2013) grouped into two functional compartments (Gandhi and Sparks, 2003; Basso and May, 2017). The superficial one has been described as consisting of three superficial layers; stratum zonale (SZ), stratum griseum superficiale (SGS), and stratum opticum (SO); which are involved in the central processing of visual information and are the targets of retinal signals (Bourne and Rosa, 2003; Markus et al., 2009). In particular, the SGS is commonly subdivided into sublayers, an upper and lower lamina (uSGS and lSGS, respectively), although their distinction, size, and complexity exhibit species-specific differences (Kaas and Huerta, 1988; May, 2006; Basso and May, 2017). Neurons in the superficial layers are considered the visuosensory division of the SC (Basso and May, 2017). In contrast, those in the intermediate (stratum griesum intermedium, stratum album intermedium) and deep (stratum griseum profundum and stratum album profundum) strata, collectively referred to as the deep compartment, are more specifically devoted to multisensory and motor functions (Casagrande et al., 1972; Harting et al., 1973; Stein et al., 1976; McPeek and Keller, 2004), earning the epithet of motor division (Basso and May, 2017).

A ubiquitous aspect of the SC is its connectivity with the retina (for a review, see Kaas and Huerta, 1988; May, 2006; Basso and May, 2017) and so distinguishing the classes of RGCs that target the SC is of particular interest (Hoffmann, 1973; Marrocco and Li, 1977; Illing and Wässle, 1981; Leventhal, 1982). Congruent with studies in macaques, the SC of marmosets receives dominant projections from parasol cells and terminals from a variety of wide-field cells, such as broad thorny, narrow thorny, smooth mono stratified, recursive, large bistratified, and tufted cells, as evidenced by bidirectional tracer injections (Kwan et al., 2018; Grünert et al., 2021). Our knowledge of the functional properties of wide-field ganglion cells that innervate the SC in the marmoset is still scarce. As expected from studies in other species of primates, electrophysiological records show that broad thorny cells are a retinal source of ON/OFF type responses in the SC (Eiber et al., 2018), as well as parasol cells input can be clearly related to the high selectivity of collicular neurons for moving stimuli (Tailby et al., 2012).

Although intraspecific variations in the proportion (Wässle and Illing, 1980; Hofbauer and Dräger, 1985; Dhande and Huberman, 2014; Ellis et al., 2016) and sublaminar arrangement of retinotectal projections (Pollack and Hickey, 1979; Conley et al., 1985) have extensively been described, the spatial profiling and delineation of these pathways remain a matter of interest. As in all mammalians studied so far, the patterns of retinal afferents in the SC of marmosets has a characteristic distribution in superficial layers (Figure 1). Bilateral retinal afferents are distributed primarily to the SGS, with dense terminals in their sublayers, and weak label in the SO and SZ layers (Kwan et al., 2018).

One of the distinctive features of the SC is a well-organized retinotopy, which is evident in all studied mammalians (Lane et al., 1971, 1973; Cynader and Berman, 1972; Kaas et al., 1974; Updyke, 1974; Ito and Feldheim, 2018). For example, in primates, the SC contains a topographic map of the contralateral visual hemifield provided by both eyes. The dorsal visual field is represented medially and the ventral visual field projects laterally. The representation of the foveal vision is located rostrally within the SC, with peripheral representation progressing caudally (Kaas and Huerta, 1988). Consistent with previous reports in macaques (Pollack and Hickey, 1979), the central retinotopic representations in the SC of marmosets demonstrate a complex pattern of retinal projections. Some areas receive binocular inputs in both the uSGS and lSGS, or contralateral input in the lSGS and binocular input in the lSGS, and others receive exclusively contralateral input (Kwan et al., 2018). Moreover, the medial and lateral colliculus exhibit overt delineation of ipsilateral and contralateral inputs in the SGS (Kwan et al., 2018). Systematic studies are needed to describe whether the same ordered representation of visual space found in the surface layers of the SC of marmosets is also present in the deep compartment of this structure.

#### 4.2.2. Accessory optic system (AOS)

In mammalians, the AOS comprises two sets of accessory fasciculi, the inferior and superior ones, and three paired terminal nuclei, the dorsal terminal, lateral terminal, and medial terminal nuclei (MTN), that receive retinal signals via the accessory optic tract (for a review, see Giolli et al., 2006; Brodsky, 2012). Different experimental approaches (Simpson et al., 1979, 1988; Clement and Magnin, 1984; Natal and Britto, 1988; Benassi et al., 1989; Lilley et al., 2018) support the functional significance for AOS in detecting signals of retinal slip and relaying them to the oculomotor circuits for image stabilization (Fredericks et al., 1988; Masseck and Hoffmann, 2009; Lilley et al., 2018). In particular, the terminal nuclei drive complementary directions of optokinetic nystagmus, albeit other oculomotor responses have been attributed to them (Simpson et al., 1979, 1988; Sun et al., 2015). The MTN and lateral terminal nuclei drive vertical optokinetic movements, while the dorsal terminal nucleus mediates the horizontal ones (Krause et al., 2014; Sun et al., 2015).

In marmosets, autoradiographic and histochemical anterograde labeling techniques revealed projections from the retina to the dorsal division of the MTN (Figure 1; Cooper and Magnin, 1986), which is congruent with anatomical studies in several species of primates (Itaya and Van Hoesen, 1983; Cooper, 1986; Weber and Giolli, 1986; Cooper and Magnin, 1987). Although retrograde tracer injections in different mammalian species showed that bistratified or gamma-like RGCs project to AOS (Farmer and Rodieck, 1982; Dann and Buhl, 1987), similar studies in marmosets are needed.

## 5. Non-image forming system

The NIF circuitry is formed by the diencephalic and midbrain nuclei, which detect environmental irradiance to modulate several physiological and behavioral processes (Daneault et al., 2016). Except for CTS, the functional significance of the NIF territories is unknown or merely speculative, a fact that contributes to its nebulous profile. In the next topic, we will discuss hodological evidence and the functional role of NIF domains in the brain of marmosets.

### 5.1. Circadian timing system

**FIGURE 2:**
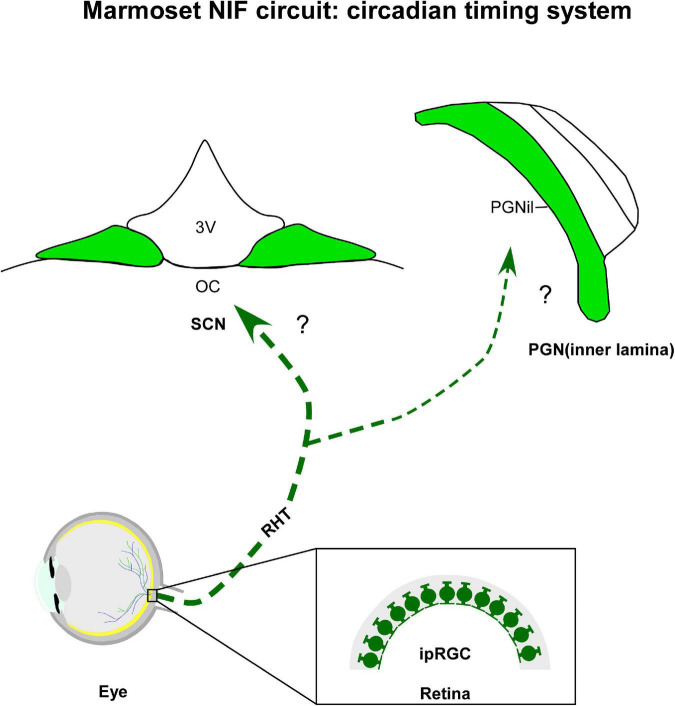
Diagrammatic representation of the marmoset circadian timing system (green). The suprachiasmatic nucleus (SCN) and inner laminar of pregeniculate nucleus (PGNil) are retinorecipient structures involved with the biological rhythms. Note that although melanopsin-containing intrinsically photosensitive retinal ganglion cells (ipRGCs) were evidenced in marmoset retina, the retinal cell type(s) providing the input to circadian centers have not yet been identified. 3v, third ventricle; oc, optic chiasma; RHT, retinohypothalamic tract.

Although the anatomically-oriented discussion is necessary for a comprehensive understanding of NIF territories, in the next section we will assemble the neuroanatomical substrate of the CTS network (Figure 2). Given its pivotal role in generating and modulating circadian rhythmicity, as well as its adaptive aspect for living organisms, including marmosets. Below, we will review the central hypothalamic components of the CTS, since systematic studies of the retinal innervation in the dorsal (DRN) and median (MnR) raphe nuclei, a discrete cluster of serotonin-containing neurons implicated in different circadian functions are absent in marmosets.

Mammalian species possess an endogenous system that synchronizes time cues, most importantly the environmental light-dark cycle, to orchestrate rhythmic biological functions, as well as ethological outputs (Xie et al., 2019; Finger and Kramer, 2021). This temporal coordination is traditionally driven by four main elements: (a) synchronizing pathways responsible for phototransduction and transmission of bioelectrical signals to a central oscillator; (b) a central oscillator, also known as the central pacemaker or master clock, a neural structure that governs circadian rhythms; (c) modulating nuclei which modify the function of the master clock and provide an indirect source of photic signaling; and (d) efferent pathways which relay timing signals to different body systems (Morin and Allen, 2006; Rosenwasser and Turek, 2015; Hastings et al., 2018). Despite this configuration being a simplified model of the CTS, a range of evidence has demonstrated that this circuitry is more complex (Zehring et al., 1984; Vosshall et al., 1994; Brancaccio et al., 2013; Mei et al., 2018). It is now clear that the CTS is a hierarchically organized network, comprising a body-wide multiplicity of circadian oscillators (extra-SCN brain clocks and peripheral clocks), in addition to cell-autonomous oscillators within virtually every cell class (Mure et al., 2018). The complexity of the circadian clock networks surpasses the purpose of this review. Recent publications (Astiz et al., 2019; Hastings et al., 2019; Finger and Kramer, 2021) approached molecular machinery, communication, and anatomy of the CTS for in-depth comprehension.

#### 5.1.1. Suprachiasmatic nucleus (SCN)

As the primary oscillator of the CTS, the SCN (Figure 2) of the anterior hypothalamus conveys temporal information, synchronizing the other clocks in the brain and body to produce coherent circadian rhythms at physiological and behavioral levels (Astiz et al., 2019). Immediately dorsal to the optic chiasm, and flanking the third ventricle (Moore and Lenn, 1972; Van den Pol, 1991), the SCN is conventionally divided into two functionally distinct domains, a ventrolateral/core and dorsomedial/shell subnuclei, distinguished by neuronal cytoarchitecture (Van den Pol, 1980; Mammen and Jagota, 2011), neurochemical phenotype (Moore et al., 2002; Morin, 2013; Allali et al., 2017), organization of afferent innervation (Moga and Moore, 1997), distribution of efferent projections (Leak and Moore, 2001), pattern of gene expression (Dardente et al., 2002), and electrical activity (Schaap et al., 2003). The functional significance of SCN compartments remains to be explored in detail, however, it is hypothesized that the prominent role of the core subregion is to maintain cellular coupling within the SCN and integrate relevant afferents for the entrainment of the master clock, while its shell subregion may have primary responsibility for coordinating the phase configuration of oscillators present in peripheral tissues and brain regions other than the SCN (Dibner et al., 2010; Welsh et al., 2010; Evans et al., 2015).

The interneuronal network of the SCN has been examined over the years. Tracing techniques have revealed the multiple neuronal connections linking the central clock with other brain territories. The foremost afferent systems of the SCN arise from the retina, IGL, pretectal complex, and the MnR (Hendrickson et al., 1972; Card and Moore, 1984; Meyern-Berstein and Morin, 1996; for a review, see Rosenwasser and Turek, 2015). At the same time, the SCN forms afferent connections with hypothalamic and extra-hypothalamic domains, allowing the adjustment of outputs from this nucleus. In addition to receiving these projections, the SCN produces diffusible signals targeting thalamic, hypothalamic, and forebrain territories (Buijs et al., 1993, 2017; Kalsbeek et al., 1993; Leak and Moore, 2001; for review see Hastings et al., 2019).

In all mammalians studied so far, the SCN receives direct photic inputs from ipRGC via the retinohypothalamic tract (RHT), a monosynaptic pathway that also innervates other NIF centers (LeGates et al., 2014). The RHT is both necessary and sufficient for photic entrainment of the SCN, as revealed by ablation, lesion, and genetic studies (Klein and Moore, 1979; Johnson et al., 1989; Panda et al., 2002). In marmosets, a dense bilateral retinal projections to the SCN have its core sub-domain as the main target, with a contralateral predominance. Sparse terminals and fibers were observed in the shell portion, specifically at intermediate and caudal levels of the SCN (Costa and Britto, 1997; Costa et al., 1998, 1999). Although the ipRGCs and their subclasses have been identified in the retina of marmosets (Ghosh et al., 1996; Jusuf et al., 2006; Szmajda et al., 2008; Masri et al., 2017), the subtypes that form the RHT remain uncertain. Further research is needed to verify this issue.

#### 5.1.2. Inner lamina of pregeniculate nucleus (PGNil)

The PGNil of marmosets (Figure 2), which lies dorsomedial to the DLG, is the probable homologous of the IGL found in the brain of non-primates. This hypothesis is classically based on the presence of the NPY^+^ cells, as well as a dense plexus of serotonergic and retinal fibers (Moore, 1989, 1993; Costa and Britto, 1997; Costa et al., 1998; Pinato et al., 2009; Lima et al., 2012) in the ventral area of the PGN. This is supported by other cytoarchitectonic (Polyak, 1957; Niimi et al., 1963; Hendrickson, 1973; Babb, 1980; Livingston and Mustari, 2000) and neurochemical evidences (Moore, 1993; Costa et al., 1998; Lima et al., 2012).

The anterograde tracer labeling shows that the PGNil receives a bilateral retinal innervation, with a contralateral predominance (Costa and Britto, 1997; Costa et al., 1998). Particularly on the ipsilateral side, a dense fiber plexus was evident when compared to the PGNol (see section 4.1.3.). Consistent with other species of primates (Moore, 1993; Théoret et al., 2000; Pinato et al., 2009), the bilateral retinal projections are concentrated in the ventral portion of the PGN of marmosets, near the P layers of the DLG, and they are sparsely distributed in the dorsal area, closer to the reticular thalamic nucleus (Costa et al., 1998; Lima et al., 2012). A comparative analysis between monkeys and humans proposes that this ventral domain would be equivalent to the IGL of rodents (Moore, 1993), a modulating nucleus, which integrates a variety of stimuli, both photic and non-photic, and transmits this consolidated information to the SCN (Sanetra et al., 2021). However, whether the PGNil neurons of marmosets show the same functional properties reported for their rodent counterpart (Dark and Asdourian, 1975; Harrington and Rusak, 1986; Cipolla-Neto et al., 1995; Goel et al., 2000; Gall et al., 2013; Sanetra et al., 2021) also needs further examination.

One open question about the retina-PGN pathway is the RGCs subtypes that innervate the PGNil. Although double immunohistochemistry for pituitary adenylate cyclase-activating polypeptide (PACAP) and CTb showed that the most ventral part of the PGN in macaques receives projections from the ipRGC (Hannibal et al., 2014), information about the typology of this retinal population, as well as hodological evidence of this connection, is still absent for marmosets.

### 5.2. Thalamus

**FIGURE 3:**
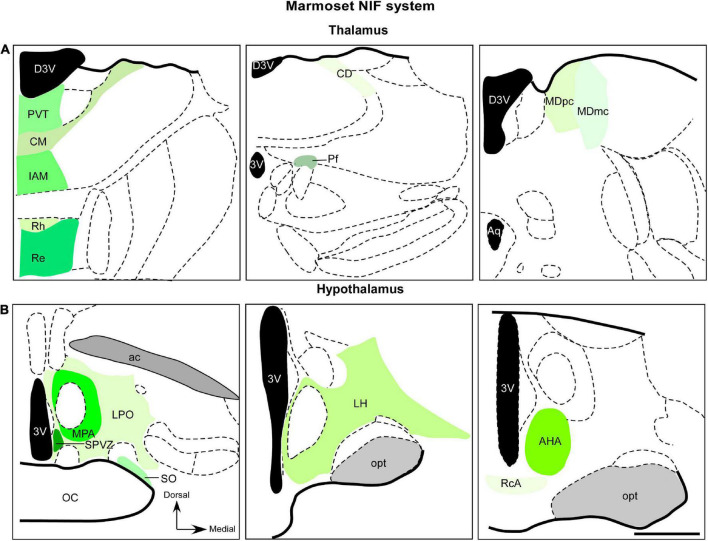
Diagrammatic representation of diencephalic marmoset non-image-foming nuclei (green). Retinal projections were described in mediodorsal nucleus, midline and intralaminar thalamic structures **(A)** and hypothalamic domains **(B)**. The RGCs subtypes that project to these regions have not yet been elucidated. 3v, third ventricle; ac, anterior commissure; AHA, anterior hypothalamic area; aq, cerebral aqueduct; CD, central dorsal nucleus; CM, central medial nucleus; D3v, dorsal 3v; Iam, inter-antero medial nucleus; LH, lateral hypothalamic area; LPO, lateral preoptic area; MDmc, magnocellular division of mediodorsal nucleus; MDpc, parvocellular division of mediodorsal nucleus; MPA, medial preoptic area; opt, optic tract; Pf, parafascicular nucleus; PvT, paraventricular thalamic nucleus; RcA, retrochiasmatic area; Re, reuniens nucleus; Rh, rhomboid nucleus; SO, supraoptic nucleus; SPVZ; sub paraventricular zone. Scale bar: 500 μm. Adapted from Paxinos et al. (2012).

Different non-image forming processes involve distinct thalamic nuclei, which form miscellaneous thalamocortical circuitry that helps to maintain homeostasis, with nociception, visceral activity, cognition, arousal, and sensorimotor activity being the most crucial functions. The mediodorsal nucleus (MD), as well as the midline and intralaminar nuclei (MIN) are key structures implicated in this functional repertory. In the next section, we will describe the retinorecipient targets in the thalamus of marmosets, with NIF properties (Figure 3A).

#### 5.2.1. Mediodorsal nucleus (MD)

The MD, also referred to as medial dorsal thalamic nuclei, nucleus medialis dorsalis, and the dorsomedial thalamus (Mitchell and Chakraborty, 2013), is a high-order thalamic relay nucleus (Guillery, 2005; Sherman, 2016) that participates in several corticosubcortical circuits, mainly those involving the prefrontal cortex (Mitchell, 2015; Golden et al., 2016). Topographically lateral to the midline nuclei and medial to the intralaminar thalamic complex, the MD is primarily involved in cognitive functions, such as learning (Gaffan and Parker, 2000; Parnaudeau et al., 2013, 2015; Ouhaz et al., 2015, 2017, 2018), odor perception (Courtiol and Wilson, 2014, 2015; Wilson et al., 2014), emotion (Timbie and Barbas, 2015), and memory processing (Funahashi, 2013), although other additional functions are suggested (Blanchard and Blanchard, 1972; Gillett and Webster, 1975; Li et al., 2004).

In primates, the MD is considered one of the largest thalamic nuclei and is cytoarchitectonically divided into at least four distinct subnuclei (Bentivoglio et al., 1993; Bachevalier et al., 1997). Despite the existence of further subdivisions, the MD domains are typically distinguished into magnocellular (MDmc), parvocellular (MDpc), caudodorsal, and lateral (Ouhaz et al., 2018). An exception to this neuroanatomical organization is described in the MD of marmosets, which is characterized by two different subregions based on cell morphology (Roberts et al., 2007), a rostromedially MDmc division, and a caudolateral MDpc one (Ray and Price, 1992; de Sousa T. B. et al., 2013). The major neural connections of the primate compartments of the MD are unique to each subregion and have been extensively summarized (Mitchell and Chakraborty, 2013; Mitchell, 2015). In addition to receiving driving inputs mainly from the prefrontal cortex (Krettek and Price, 1977; Goldman-Rakic and Porrino, 1985; Groenewegen, 1988; Ray and Price, 1993; McFarland and Haber, 2002; Xiao et al., 2009), the distinct portions of the MD have differential connectional patterns with areas of the medial temporal lobes (perirhinal and entorhinal cortex and the amygdala (Krettek and Price, 1977; Aggleton and Mishkin, 1984; Aggleton et al., 1986; Russchen et al., 1987; Groenewegen et al., 1990; Saunders et al., 2005), as well as the cingulate cortex, insular cortex, and supplementary motor cortex (for a review, see Mitchell and Chakraborty, 2013; Mitchell, 2015; Ouhaz et al., 2018). Furthermore, the MD is a target of modulatory inputs from the pallidum, the reticular thalamus, midbrain, and brainstem regions (Kuroda and Price, 1991a,b; Sherman and Guillery, 1996), structures particularly related to ocular movements, such as the substantia nigra pars reticulata (Wurtz and Goldberg, 1972; Harting et al., 1980; Ilinsky et al., 1985; Russchen et al., 1987) and the motor layers of the SC (Erickson et al., 2004).

The retinal afferents in the MD of marmosets was revealed by anterograde tracer histochemistry (de Sousa T. B. et al., 2013). This work showed an exclusive retinal contralateral innervation, with sparse retinal arbors and terminals into MDmc and MDpc subnuclei (Figure 3A) in the caudal aspect of the MD. Furthermore, retinal fibers oriented dorsoventrally, and detailed morphology of the retinal axons were described, including simple endings, large caliber axons with numerous varicosities, and rosette-like clusters (de Sousa T. B. et al., 2013). The retina-MD pathway has also been described in rock cavy (*Kerodon rupestris*), as revealed by an anatomical study, although this innervation is restricted to the medial parts in the mid and caudal levels of this nucleus (Nascimento et al., 2010).

Although electrophysiological (Schlag and Schlag-Rey, 1986; Tanibuchi and Goldman-Rakic, 2003; Wyder et al., 2003; Tanaka, 2007) and anatomical studies (Wurtz and Goldberg, 1972; Kievit and Kuypers, 1977; Goldman-Rakic and Porrino, 1985; Russchen et al., 1987) indicate the MD participates in visuomotor integration in primates (Wurtz and Albano, 1980; Tanaka, 2007), the functional role of the retinal-MD circuit remains unexplored. It is speculated that the retina-MD projection potentially provides an indirect route from the retina to the prefrontal cortex, whose photic input might exert a specific influence on prefrontal cortical functioning (de Sousa T. B. et al., 2013). Furthermore, substantial research is needed on the issue of the RGCs subtypes that innervate the MD.

#### 5.2.2. Midline and intralaminar nuclei (MIN)

The MIN are a higher-order nuclear complex, which was initially thought to be a non-specific arousing circuit in the brain due, among other features, to their widespread connectional pattern with the cortex (Bentivoglio et al., 1991; Saalman, 2014; Zhou and Zhu, 2019). Anatomical and functional data have demonstrated that the MIN are involved in specific brain functions, from cognitive to sensorimotor properties (Bentivoglio et al., 1991; Groenewegen and Berendse, 1994; Van der Werf et al., 2002; Vertes et al., 2022). Furthermore, the growing electrophysiological evidence supports the functional role of the MIN in the control of the transmission of cortical information (for a review, see Saalman, 2014). Due to space limitations, we will not discuss the architecture, connectivity and functions of the marmoset MIN, and will confine this review to retinothalamic projections. Previous studies (Bentivoglio et al., 1991; Groenewegen and Berendse, 1994; Van der Werf et al., 2002) provide a well-documented characterization of the MIN.

Anterograde labeling from the marmoset retina has revealed a moderate plexus of retinal fibers, forming a “continuum” in the dorsoventral direction (Figure 3B). This innervation starts from the paraventricular nucleus (PVT), reaching the inter-antero-medial reuniens and rhomboid nuclei. In the intralaminar complex, a sparse terminal plexus was found contralaterally, in the central dorsal nucleus. The central medial and parafascicular nuclei also exhibited scattered terminal fibers (Cavalcante et al., 2005).

As far as we know, except for the rock cavy, the retinal afferences to the MIN have not been reported in any vertebrate species. Only the PVT receives a direct retinal projection in the rock cavy (Nascimento et al., 2008). Under these circumstances, it is easy to suppose that retinal innervations in the MIN of marmosets, except for the PVT, are a species-specific characteristic. However, anatomical and evolutionary studies in other species are needed to verify the possible universality of these afferences and elucidate their functional significance. Moreover, it is important to stress that both were studies performed the anterograde transport of CTb, a tracer extensively used for monosynaptic mapping (Lai et al., 2015). Therefore, it is unlikely that the labeled CTb-fibers/terminals described in those works could be due to the transsynaptic transport from other retinorecipient domains (see comments in Costa et al., 1999). Furthermore, the CTb immunoreactive elements were not observed in well-stablished secondary visual areas, such as the visual cortex (Cavalcante et al., 2005; Nascimento et al., 2008), which corroborates the evidence. Surely, specific functional and evolutionary work is needed regarding the participation of retinal projections in the functional and phylogenetic aspects of the MIN. Further studies on the class of RGCs that innervates the MIN of marmosets are also required since this specific cell population was not yet characterized.

One interesting aspect to be considered is the retina-PVT pathway identified in the marmoset brain (Figure 3B). PVT is the main component of the midline thalamic nuclei, which extends rostrocaudally and ventral to the third ventricle (Kirouac, 2015). This nucleus is considered a hub of neural circuits underlying drug addiction, anxiety, emotional processing, and defensive responses (Zhou and Zhu, 2019; Barson et al., 2020; Kirouac, 2021). However, it is suggested that the PVT also takes part in the circadian regulation, based on lesion studies (Bhatnagar and Dallman, 1999; Moga and Moore, 2000; Salazar-Juárez et al., 2002). The fact that the PVT receives input from CTS structures, including IGL, DRN, and MnR nuclei (Cornwall and Phillipson, 1988; Moga and Moore, 2000; Leak and Moore, 2001; Li and Kirouac, 2012), as well as reciprocal connections with the SCN (Moga et al., 1995; Vertes and Hoover, 2008) also indicates that it may be involved in functions related to the modulation of circadian rhythms. This is in line with the view that the neural activity of PVT is enhanced during the active phase of the light cycle (Peng et al., 1995; Novak and Nunez, 1998; Kolaj et al., 2012). Since this structure has been hypothesized to be involved in circadian modulation, PVT neurons may be the centers of regulatory circuits of the sleep-wake cycle and circadian system (see comments in Colavito et al., 2015). Nevertheless, functional properties and phylogenetic evidence of the retina-PVT pathway require further research.

### 5.3. Hypothalamus

The NIF hypothalamic network in the marmoset brain comprises extra-SCN nuclei involved in control of many fundamental processes, from circadian rhythms to reproductive behaviors. In this section, we will highlight evidence from retinal innervation, as well as discuss the possible functional aspect of this NIF circuitry.

#### 5.3.1. Extra-SCN regions

Although the SCN is a well-known hypothalamic target of the retina, hodological studies demonstrated retinal projections to other domains of this structure in several mammalian species (Pickard and Silverman, 1981; Johnson et al., 1988; Murakami et al., 1989; Levine et al., 1991; Youngstrom et al., 1991; Tessoneaud et al., 1994; Abizaid et al., 2004; Hattar et al., 2006). These nuclei play different NIF functional roles, from circadian rhythmicity to reproductive behavior (Saper and Lowell, 2014). In marmosets, diffuse retinohypothalamic projections were described in lateral and medial preoptic, anterior hypothalamic, lateral hypothalamic, and retro chiasmatic areas, besides the supraoptic nucleus, and subparaventricular zone, as revealed by tract-tracing procedures (Costa et al., 1999; Figure 3B). This latter structure is known as a critical hypothalamic hub for driving rhythmic output from SCN and ultimately modulate circadian rhythms of a several physiological process (Saper, 2013; Vujovic et al., 2015; Wu et al., 2018). A comprehensive overview of these hypothalamic regions in mammalians is beyond the scope of this review and a detailed discussion of these nuclei can be found within classical (Moore and Lenn, 1972; Moore, 1973) and recent works (Canteras et al., 2011; Morin and Studholme, 2014). Here, we will focus on retinal innervation and the functional significance of these afferences.

Costa et al. (1999), via the analysis of anterograde tracing, showed that several hypothalamic areas receive retinal projections, particularly those involved in many distinct light-mediated behaviors, such as sleep, body temperature, circadian rhythm phase control, and neuroendocrine processes related to reproductive functions (Costa et al., 1999). However, there are no functional studies of these retinohypothalamic projections. The organization of RGCs that innervate the hypothalamic extra-SCN regions has also not been researched in marmosets.

### 5.4. Midbrain

Retinal afferents innervate a restricted cell grouping in the midbrain, which exhibits NIF functional characteristics, such as pain responses (Tracey et al., 2002; Loyd and Murphy, 2009), defensive and aversive behaviors (De Oca et al., 1998; Benarroch, 2012), central autonomic control (Saper and Stornetta, 2015), and modulation of circadian rhythms (Ciarleglio et al., 2011; Whitney et al., 2016). In a variety of species, retinal inputs have been described in the periaqueductal gray (Fite et al., 1999), DRN (Foote et al., 1978; Kawano et al., 1996; Reuss and Fuchs, 2000), and parabrachial complex (PBN) (Fite and Janusonis, 2002). It is important to explain that, although the periaqueductal gray plays a critical role in neurovegetative functions and behavioral responses to threatening stimuli (Faull et al., 2019), its retinal innervation is yet to be determined in marmosets. Furthermore, as previously mentioned, there is no hodological evidence in marmosets demonstrating the retina-DRN pathway. Thus, these factors restrict the explanation of the retinal input to the PBN, a hub for autonomic functions, and interoceptive and exteroceptive inputs relevant to sensory processing (Chiang et al., 2019).

#### 5.4.1. Parabrachial complex (PBN)

**FIGURE 4:**
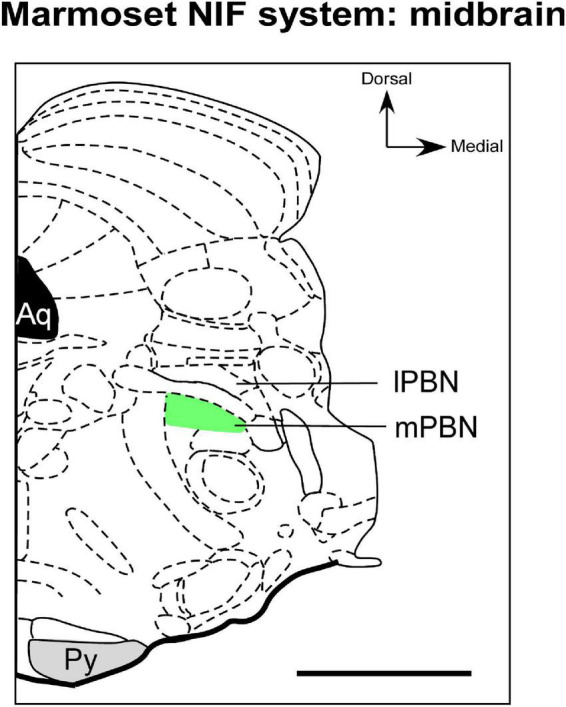
Schematic representation of the parabrachial complex in coronal section of the marmoset brain. The types of RGCs that send afferents to mPBN (green), a NIF site in midbrain, have not yet been documented in marmoset. aq, cerebral aqueduct; lPBN, lateral parabrachial nucleus; mPBN, medial parabrachial nucleus; Py, pyramidal tract. Scale bar: 500 μm. Adapted from Paxinos et al. (2012).

In most mammals studied to date, the PBN has been described as a cell cluster in the dorsolateral pons, which is dissected by the superior cerebellar peduncle into two distinct subnuclei, the medial parabrachial (mPBN) and lateral parabrachial (lPBN) nuclei (Fulwiler and Saper, 1984; Hansell and Frank, 1991; Chiang et al., 2019; Figure 4). However, anatomical studies in mice, cats, and monkeys demonstrated a third subdivision of the PBN, the Kölliker–Fuse nucleus, a collection of neurons located in the ventrolateral region of the superior cerebellar peduncle (Saper and Loewy, 1980; Fulwiler and Saper, 1984). In marmosets, the PBN is formed by mPBN and lPBN, based on cytoarchitectonic data (Engelberth et al., 2008).

As the interface between the medullary reflex control and the behavioral and integrative regulation of the central autonomic network, the PBN has long been recognized as a pivotal structure in autonomic control (Saper and Stornetta, 2015). Traditionally, sensory input relevant to taste is processed by the mPBN, and viscerosensory information (visceral malaise, itch, blood pressure, hydric ingestion, and sodium appetite) has been consistently correlated with the lPBN activity (Hugelin and Vibert, 1974; Hansell and Frank, 1991; Reilly, 1999; Davern, 2014; Menani et al., 2014). Furthermore, its functional role in processing nociceptive and thermosensory stimuli has been revealed in electrophysiological, optogenetic, and behavioral approaches (Yahiro et al., 2017; Barik et al., 2018; Xu et al., 2019; Sun et al., 2020).

This functional complexity is based on the hodological pattern of the PBN (Gioia et al., 2000). Retrograde and anterograde tract tracing both revealed that the PBN is the target of axonal inputs primarily from the nucleus tractus solitarii (Ricardo and Koh, 1978; Tokita et al., 2009) as well as trigeminal and spinal dorsal horns projections (Cechetto et al., 1985; Hylden et al., 1985). Other connectional studies demonstrated that the PBN innervated by several areas of the brain, such as the ventral thalamus, insular cortex, limbic cortex, central nucleus of the amygdala, bed nucleus of the stria terminalis, and hypothalamus (Saper and Loewy, 1980; Fulwiler and Saper, 1984; Bernard et al., 1993; Bester et al., 1997; Krout and Loewy, 2000; Grady et al., 2020).

Regarding retinal innervation, retrograde labeling techniques show that there is discrete and exclusive retinal input in the mPBN of marmosets (Figure 4; Engelberth et al., 2008). This pattern is different from all mammalians studied so far, in which their projection appears to involve the lPBN (Fite and Janusonis, 2002). It is speculated that this hodological variation in retinal innervation of the PBN of marmosets could have a functional partition, a characteristic that rodents apparently do not exhibit (Engelberth et al., 2008). To our knowledge, no comparative or phylogenetic study evidenced whether the retina-mPBN circuitry is a general primate attribute or just a species-specific feature. Furthermore, the functional role of the retina-PBN pathway has also not been clarified in marmosets. Engelberth et al. (2008) suggest that this connectional pattern may represent a photic integration node and viscerosensory stimuli to modulate visual processing.

## 6. Conclusion

We aimed to demonstrate the current state of knowledge on the IF and NIF circuitry of marmosets. The most studied structures of IF processing, in marmosets and other primates, are the DLG, PI, and SC. One the other hand, the SCN is a well-characterized NIF domain in all animals studied so far. The evidence, considered here for these nuclei, supports considerable progress made in understanding the retinal connectivity of marmosets in the past decades. Consequently, it can be considered an excellent non-human primate model to investigate the anatomy and function of the IF and NIF systems.

Besides the regions of intense research interest mentioned above, our knowledge regarding the IF and NIF networks in marmosets remains incomplete. In the case of IF midbrain structures; such as the pretectal complex; our limited knowledge reflects, in part, the difficulty in delineating the cytoarchitectonic boundaries of these nuclei and the fact that few publications describe the presence of retina-PTC pathways. Regarding NIF territories, the functional properties and phylogenetic significance of the retinal innervation in the mPBN and MIN of the marmoset are uncertain. Further comparative work is needed to solidify knowledge regarding the function and universality of these pathways.

It is important to note that little is known about the functionality and RGC types that innervate the retinorecipient nuclei of marmosets. Although the wide-field RGC classes have been reported by their projections to DLG, PI, and SC, their functional aspect remains opaque. In the case of all NIF territories, the retinal population is uncertain or merely hypothetical, particularly related to the function of the involved system, such as the CTS. Substantial morphological retinal studies combined with hodological techniques are needed to draw conclusions regarding the origin of retinal fibers in the NIF domains.

The two last decades have seen a rapid advancement in the establishment of robust protocols for viral tracers, computational pipelines, structural MRI, functional MRI, and genetic modifications among other important developments. We believe that these approaches could reveal the precise functional and connectional organization of retinorecipient areas in all species of vertebrates, including marmosets, with three-dimensional reconstruction of their retinal axonal projections and targets, from fetal to all aging levels.

Finally, we have noted that, although retinal connectivity has been a prominent focus for hodological research for years and impressive progress has been made in understanding its functionality, pivotal information is still absent, as mentioned throughout this review. These issues represent the next set of challenges for keeping this field relevant and for building essential tools to comprehend IF and NIF functions.

## Author contributions

NS, ES, and RE conceived and wrote the manuscript with support from EN, MC, and SS. JC prepared and revised the manuscript critically. All authors contributed to the article and approved the submitted version.
